# SymbioGenomesDB: a database for the integration and access to knowledge on host–symbiont relationships

**DOI:** 10.1093/database/bav109

**Published:** 2015-11-25

**Authors:** Mariana Reyes-Prieto, Carlos Vargas-Chávez, Amparo Latorre, Andrés Moya

**Affiliations:** ^1^Institut Cavanilles de Biodiversitat i Biologia Evolutiva, Universitat de València, Calle Catedrático José Beltrán 2, 46980 Paterna, València, Spain,; ^2^Fundación para el Fomento de la Investigación Sanitaria y Biomédica de la Comunitat Valenciana (FISABIO)-Salud Púbica, Avenida de Catalunya 21 46020, València, Spain and; ^3^CIBER en Epidemiología y Salud Pública (CIBEResp), Madrid, Spain

## Abstract

Symbiotic relationships occur naturally throughout the tree of life, either in a commensal, mutualistic or pathogenic manner. The genomes of multiple organisms involved in symbiosis are rapidly being sequenced and becoming available, especially those from the microbial world. Currently, there are numerous databases that offer information on specific organisms or models, but none offer a global understanding on relationships between organisms, their interactions and capabilities within their niche, as well as their role as part of a system, in this case, their role in symbiosis. We have developed the SymbioGenomesDB as a community database resource for laboratories which intend to investigate and use information on the genetics and the genomics of organisms involved in these relationships. The ultimate goal of SymbioGenomesDB is to host and support the growing and vast symbiotic–host relationship information, to uncover the genetic basis of such associations. SymbioGenomesDB maintains a comprehensive organization of information on genomes of symbionts from diverse hosts throughout the Tree of Life, including their sequences, their metadata and their genomic features. This catalog of relationships was generated using computational tools, custom R scripts and manual integration of data available in public literature. As a highly curated and comprehensive systems database, SymbioGenomesDB provides web access to all the information of symbiotic organisms, their features and links to the central database NCBI. Three different tools can be found within the database to explore symbiosis-related organisms, their genes and their genomes. Also, we offer an orthology search for one or multiple genes in one or multiple organisms within symbiotic relationships, and every table, graph and output file is downloadable and easy to parse for further analysis. The robust SymbioGenomesDB will be constantly updated to cope with all the data being generated and included in major databases, in order to serve as an important, useful and timesaving tool.

**Database URL**: http://symbiogenomesdb.uv.es

## Introduction

Symbiotic relationships are ubiquitous in our planet. They happen within every niche observable in our world, even within our bodies, and they are crucial in the maintenance of every ecosystem. Although the term symbiosis is sometimes confounded with mutualism, this intimate association can also be parasitic or commensal (1).

Generally, symbiosis occurs between a eukaryotic partner forming this close relationship with one or multiple species of bacteria. Furthermore, the number of sequenced genomes is growing rapidly, especially in the microbial world (2). For this reason, the amount of information has become so immense, that we need to find better ways to make it comprehensive and useful. The aim of SymbioGenomesDB is to facilitate research through the gathering of information from organisms involved in symbiotic relationships.

Initially designed to comprise all the newly sequenced and annotated genomes of endosymbionts of insects, SymbioGenomesDB has grown to include all the bacteria and even some eukaryotes, which are known symbionts worldwide. The catalog of these symbionts includes just above 1050 genomes, while the list of hosts comprises 216 organisms, most of them Eukaryotes.

The central mission of SymbioGenomesDB is to host and support the growing and vast symbiotic–host relationship information, to uncover the genetic basis of such associations. As a highly curated and comprehensive systems database, SymbioGenomesDB provides web access to this complete catalog of fully sequenced and annotated genomes of symbiotic organisms and their features, including genetic and genomic sequences, their genomic characteristics and the symbiotic interaction in which they participate, as well as links to the central database NCBI (http://www.ncbi.nlm.nih.gov/) (3). SymbioGenomesDB will be routinely updated to keep up with the growth rate of genomic sequences available, in order to serve as a recognized authority and a comprehensive data integration site and repository for symbionts’ genetic, genomic and phenotypic data, derived from major data providers.

## Data collection

**Figure 1.**
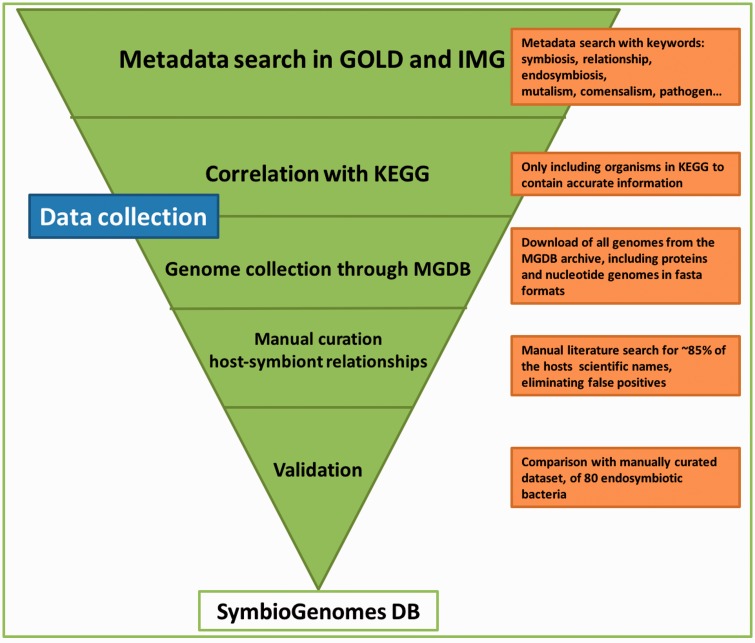
Schematic representation of the data collection workflow. The graphic illustrates the mayor modules of the data collection and the action taken in each step. The pipeline is highly tunable, and every update will be easier and shorter, since this first manual curation has been so thorough. The triangle shape is meant to convey that each step acts as a filter of data we are not interested in, until we end up with the highly curated catalog that SymbioGenomesDB currently offers.

The workflow we followed for the collection of the data is depicted in Figure 1. Furthermore, the steps in the figure are thoroughly explained next:

1. First, we created a list of all of the symbiotic relationships denoted in GOLD (http://www.genomesonline.org/) (4) and IMG (https://img.jgi.doe.gov) (5), through metadata searches. Both are highly curated databases that contain every registry of all genomes available up to date and those that are in progress, with highly curated and detailed metadata.
2. Next, we correlated the aforementioned list of symbionts to the genomes available in KEGG (6, 7) to have the most information of each genome. We only included those genomes that are classified as finished or permanent drafts, to get the most complete set of information on each genome.
3. Following, we downloaded the genetic information of the genomes included in the Microbial Genomes Database archive (8), and cross referenced it to our list of symbiotic relationships.
4. We also completed the host field as an association to each symbiont with the metadata from GOLD and IMG, although most of the cases (∼80%) did not have a host associated. We manually curated the link between host–symbiont of all of the organisms without a host through literature searches.
5. This catalog was then validated when compared to a small dataset of ∼80 endosymbiotic bacteria of insects we had manually curated in our lab (unpublished data) which we achieved based on literature, and found every relationship in accordance.

## Database architecture and web interface

SymbioGenomesDB consists of an html front web interface, and the database itself is built using custom R (9) scripts for the integration of data and parsing of the genomes for the database, coupled with the R Package Shiny from RStudio (10). Shiny is a powerful web framework that allows us to build interactive web applications with R. It incorporates the computation power of R with the interactivity of web pages. Applications built with Shiny respond automatically as users type their queries and ask for their outputs by pressing buttons. Thus, when new queries are written into SymbioGenomesDB, the organisms, genomes or genes searched for are automatically displayed on the screen. Also, for each genome, the size and metrics such as gene number and GC content, are automatically computed and shown as output. Users with no experience in programing languages can use the database without difficulty. The html web page was designed with Sandvox for Mac, and the server is published in the symbiogenomesdb.uv.es server hosted by the University of Valencia. SymbioGenomesDB is freely accessible at http://symbiogenomesdb.uv.es.

## Database features

**Figure 2.**
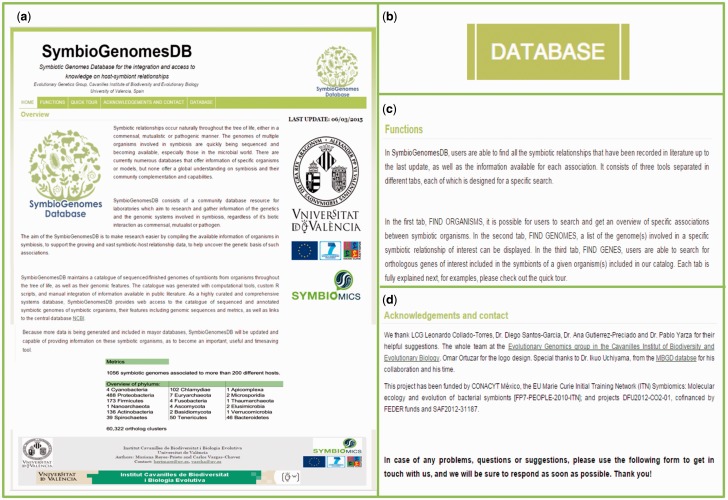
Database overview. In HOME (a), there is a complete overview of the importance of the database and its purpose in detail. The line of buttons in the above green menu, as well as the menu on the right, denote the different parts of the web interface, with special importance to the button (b) “Enter Database”, which will open the database in Shiny from the R Studio. An explanation of the functions (c) of the database is also included, as well as a quick tour explained in detail throughout this article, and the Acknowledgments (d) for the support of this work.

We have designed a user-friendly web interface for our database. Upon entrance, users will find the welcome page and the information to understand the full use and capabilities of the database, as well as everything it has to offer (Figure 2).

As aforementioned, in SymbioGenomesDB, users are able to find all the symbiotic relationships that have been recorded in literature, as well as the information available for each association. It consists of three tools separated in different tabs, each of which is designed for a specific search. In the first tab, FIND ORGANISMS, it is possible for users to search and get an overview of specific associations between symbiotic organisms. In the second tab, FIND GENOMES, a list of the genome(s) involved in a specific symbiotic relationship of interest can be displayed. In the third tab, FIND GENES, users are able to search for orthologous genes of interest included in the symbionts of a given organism(s) included in our catalog. These tools are fully described in the next section.

## Find organisms

**Figure 3.**
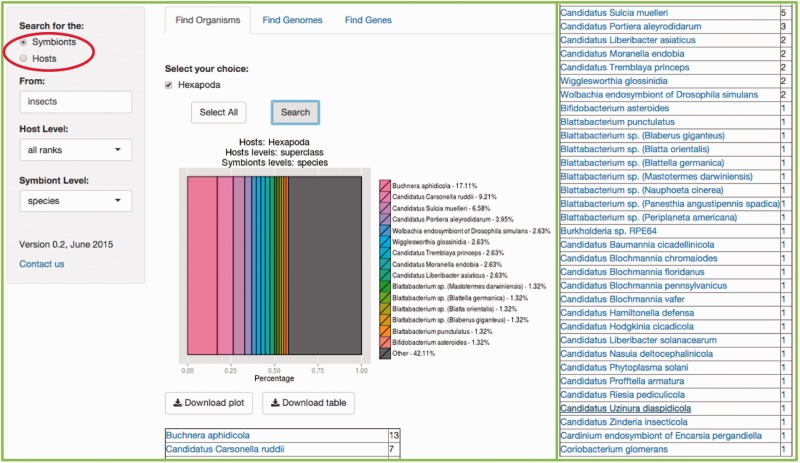
Example of the *Find Organisms* tab. In our lab at the University of Valencia we work with insect symbiosis (11, 12, 13). This example shows the result of searching for the symbionts of ‘insecta' with the default ‘all ranks' host level, and the ‘species' level selected for symbionts. Be aware that the matching result indicates that the host level of the query is *class,* since it is where a match was found. The results include an abundance graph including the first most representative 14 matches, plus a summary of the rest of the matches, and a table of organisms matching the users query, and the number of matches for each specie, phylum, class or whichever taxonomy level explored. The rest of the resulting table is at the right of the figure for space limitations.

In this tab, users are able to search for a specific symbiotic association of interest, by entering the common name, the species scientific name, the genus, the class or any taxonomy level keyword based on NCBI’s taxonomy. Users are also encouraged to enter any random organism they can think of, to explore and get a better understanding of the searches SymbioGenomesDB does, including shuffling between the taxonomic levels, since this a useful way to start any type or analysis of these organisms. Users can explore through broad searches, from wider taxonomy levels, such as Kingdom or Families, to narrow searches, to the level of species and strains. Figure 3 is an example of a specific search of our interest.

It is important to denote which partner of the symbiosis will be searched for. It can either be the symbionts of your host of interest, or the other way around, and selecting the proper circle available indicates it (Red rectangle in Figure 3).

Selection of the host(s) taxonomy level is available in the scrolling menu below the search bar (the default ‘all ranks’ will search in every taxonomic level and as a result, show the level where the match is found). In the same manner, a selection the symbiont(s) taxonomy level of interest is also available (the default ‘all ranks’ is equal in every search). Typically, results of the query entered will be shown in just milliseconds. Searching for an organism with a large amount of associated symbionts (e.g. human or pig, since there are a lot of pathogenic bacteria associated with these species), can take a few seconds.

Both the resulting plot and resulting list of organisms can be downloaded. Also, the resulting list of organisms (in Figure 3, all the species involved in symbiotic relationships with insects) includes links to their respective specie at NCBI Taxonomy.

## Find genomes

**Figure 4.**
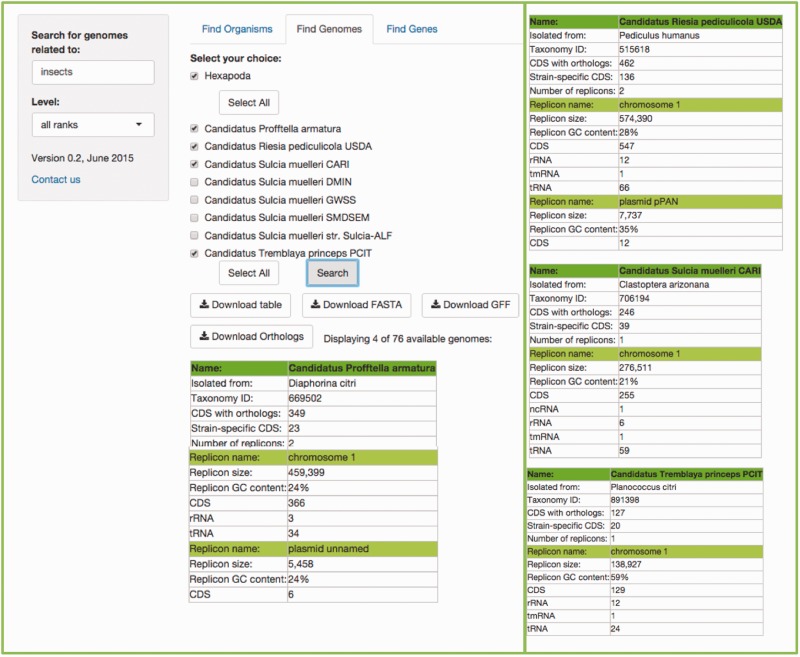
Example of the *Find Genomes* tab. The result of searching for the genomes associated to ‘insects' with the default ‘all ranks’ taxonomic level. First, users get a scrolling menu from which they can select genomes of interest, or all genomes available in this search, which in turn displays a table which shows the names of the organisms selected in the scrolling menu, the precise host with which the symbiotic relationship exists, as well as metrics and characteristics of their genomes. The rest of the resulting table is at the right of the figure for space limitations.

This search will retrieve the genomes associated with a specific host. Users can write the common name, the scientific species name, the class, the genus, etc., of their genome(s) of choice according to the taxonomical level of interest, in the search box (the default ‘all ranks’ will search in every taxonomic level). Figure 4 shows an example.

In this case, the search can be done for a specific symbiont, or a specific host. If the search is for a host, another menu listing all its symbionts’ genomes will become available, where the genomes of interest can be selected.

In a matter of seconds, tables consisting of the metrics (genome size, NCBI's taxon id, CDSs, GC content, etc) of each genome selected, the symbionts’ host, plasmids and all chromosomes, in cases where more than one chromosome is present, will be shown.

An important feature embedded in this search is that users can download a table with all of the orthologous genes from the genomes they have searched for (at least two), with just one click of a button. This table can be easily parsed for further analysis. The complete set was obtained from the MBGD: Microbial Genomes Database (http://mbgd.genome.ad.jp) (8). This orthology table includes six different fields of information for each orthology found, including the gene name of each ortholog, the description of the gene, their id in four different databases, including MBGD, COG (14), KEGG and TIGR (15). Then, each organism of the search is displayed as a column (abbreviated with their KEGG organism id as the header of the column), with a list of each ortholog shared with at least one other organism in the subset selected. The table(s), the FASTA file(s) and/or the GFF file(s) of the organisms resulting of the search are also available for download.

## Find genes

In this tab, users can search for genes in any symbiont present in our catalog, or a table of orthologous genes included in two or more genomes of interest, involved in symbiosis. These orthologs have been calculated with several algorithms, according to the MBGD: Microbial Genomes Database (8).

**Figure 5.**
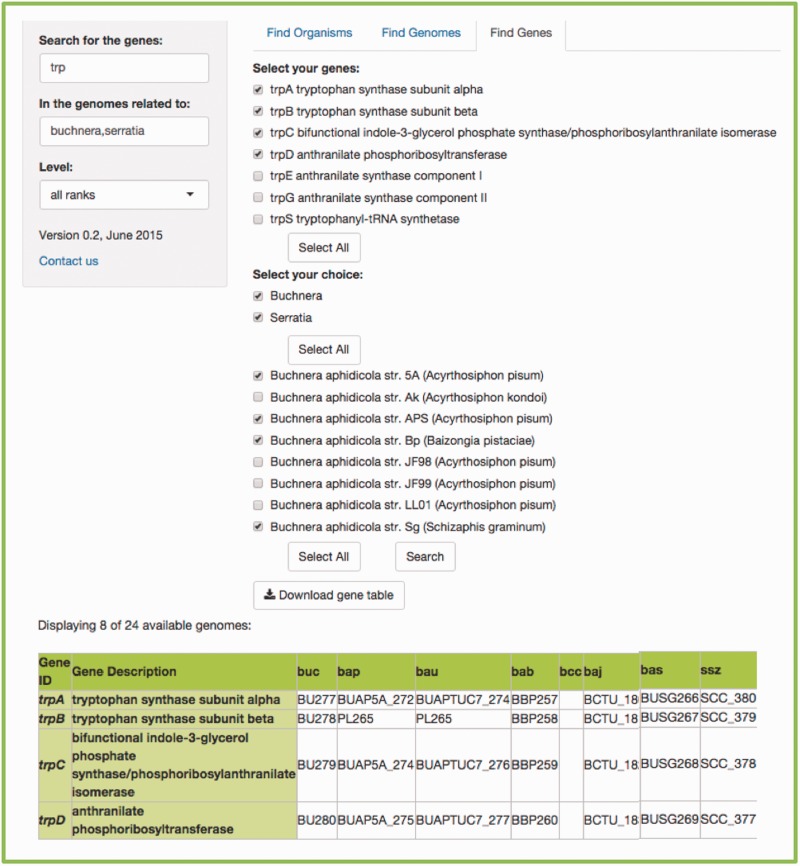
Example of a *FIND GENES* search. (a) Searching for all the genes included in the tryptophan biosynthesis in the genomes related to insects. We get two scrolling menus and selected all the tryptophan genes and the genomes of several species of the *Buchnera* genus, as well as the species *Serratia symbiotica*. (b) The resulting table that lists the orthologs found between the genomes we selected, including the bacteria working as cosymbionts in the aphid *Cinara cedri*, that participate in an exceptional metabolic complementation of the tryptophan metabolic pathway (16). (c) Even though the table shows the abbreviated genome names from KEGG (6, 7), if you scroll over the name, you get the complete species name in a little box below the pointer.

Users need to write the gene of interest in the first search bar. If more than one gene is searched for, users must use commas to separate searches. In the second search bar, the common name, the scientific name, the class, the genus, etc., of the genome(s) of interest according to the taxonomical level of choice, must be written (the default ‘all ranks’ will search in every taxonomic level). As a result, two menus will be available, to select the gene(s) and genome(s) of interest, accordingly. The search can be done for a specific symbiont, or a specific host. If searching for a host, another menu listing all its symbionts’ genomes becomes available. Figure 5 shows an example.

The resulting table will be a table including the genes and the genomes selected in the menus. Every output is available for download, as flat files for further and easy parsing and analysis.

## Discussion and future directions

The boom in microbial genomes publications has necessitated the development of several tools to categorize and better organize the data we are given, to fully appreciate it for analyses and gain as much knowledge as possible from it. As a highly curated and comprehensive systems database, SymbioGenomesDB provides access to the complete catalog of fully sequenced and annotated genomes of symbiotic organisms and their features including genomic sequences and metrics, which can be useful for research, reducing the time-consuming search for biotic relationships and the features of the organisms involved.

SymbioGenomesDB is a core component of an extensive set of genome informatics resources that comprises several microbiology databases. It is linked to NCBI (3), and these systems conjoined will provide an intensively integrated and accessible data resource representing the highest quality and most comprehensive consensus and experimental views of host–symbiont relationships as experimental subjects.

Furthermore, SymbioGenomesDB allows users to search for specific sets of genes, which can also be useful when working with an organism(s) metabolism, comparative genomics, complementation within organisms, etc. We are open to implementing more features if requested by users.

Finally, we foresee that the continued development of high-throughput sequencing technologies and their dropping prices will result in the sequencing of numerous new organisms involved in symbiotic relationships. As more data is generated and published, SymbioGenomesDB will be updated and therefore, capable of providing information on the genomic features and the sequences of the organisms involved.
